# A Summer Research Experience to Increase Diversity in Healthcare: a 4-Year Follow-up Study

**DOI:** 10.1007/s40670-023-01940-7

**Published:** 2023-11-10

**Authors:** Behnoosh Afghani, Herschell Valenova Dayag

**Affiliations:** 1 Department of Pediatrics, UC Irvine, Orange, CA USA; 2 Children’s Hospital of Orange County, Orange, CA USA; 3 University of California, Irvine, CA USA

**Keywords:** Summer research program, Mentorship, Pipeline, Underrepresented, First-author publications

## Abstract

This report describes the educational follow-up of the college coaches who participated in our Summer Research Programs from 2012 through 2019. Our program was successful as all the 45 college coaches submitted a total of 54 abstracts to a regional conference, and 100% of them were accepted for publication. On follow-up in 2023, most of the college coaches, including women and those from minority backgrounds, were enrolled or graduated from a health professional school or worked in a healthcare setting. Despite our small study population, our research program can serve as a model to increase diversity in healthcare and science fields.

## Introduction

Although the proportion of female and minority enrollment in healthcare-related professional schools has increased over the past few decades, there continues to be an underrepresentation of women and minorities engaged in medical research and academic medicine [1, 2]. The Association of the American Medical Colleges (AAMC) remains committed to ensuring access to medical-related careers for individuals from racial/ethnic underrepresented minority (URM) groups: Blacks, Mexican Americans, American Indians, Alaska Natives, and Native Hawaiians [3]. Furthermore, the first and last authors of publications in high-impact academic medicine journals are less likely to be female or from underrepresented minority (URM) backgrounds [4, 5]. During college, female and minority students are more likely to perceive research negatively and participate in research [6]. A lack of sense of belonging [7, 8] and absence of strong mentors and role models have underscored why females and minorities leave science, technology, engineering and mathematics (STEM) majors [9]. Summer research programs have emerged as a promising means of increasing enrollment in healthcare and science fields [4, 10, 11]. However, studies that focus on follow-up of the participants are limited.

In this article, we share insights about an inclusive and innovative summer research program at the University of California, Irvine School of Medicine, with a vision of increasing the proportion of underrepresented students in health sciences. Our main objective was to stimulate the students’ interest at various levels in research with the goal of first authorship in abstracts submitted by college students. Our secondary goal was to provide long-term mentorship to empower and assist college students in accomplishing their goals in pursuing healthcare or science-related professions. We present the follow-up of college coaches at least 4 years after their matriculation in our program.

## Activity

During the past 15 years, a variety of health and enrichment programs were created at University of California, Irvine School of Medicine, with a vision of helping students achieve their goal of pursuing a career in healthcare. The cascading nature of our programs has been described previously [12, 13]. In brief, college and medical students coach a group of high school students under the direction of the faculty leader. In this paper, we describe the evolution of the research program and the follow-up of college coaches.

### On-Site Research Program

In 2012, we started a 2-week on-site Research Program where groups of high school students and college coaches rotated in different laboratories and got a sense of translational science research under the mentorship of faculty and Ph.D. students and college coaches. The students and coaches completed modules related to human subject research, research ethics, and animal and lab safety before attending the labs. In the afternoons, the participants spent their time in various laboratories and, through hands-on experience, learned about in vitro lab techniques such as immunostaining, nanotechnology, electroencephalogram, and electrophoresis. Additionally, they became familiar with different in vivo lab techniques through animal models and the importance of “bench-to-bedside” research. In the mornings, the high school students worked on a literature review project under the direction of their coach.

### Online Research Program

In 2015, we started an additional 3-week summer online research program where high school students and their college coaches learned about the basics of research and did a literature review project. Through virtual sessions, the students learned about human subject research, research ethics, health disparities, basic statistics, and how to do a literature search.

### Literature Review Project

For both the on-site and online programs, the high school students were divided into groups, and under the mentorship of their college or medical student coach, they performed a literature review on a topic. They learned how to form a hypothesis, use various reputable biomedical search engines, collect data, and write their results and conclusion in a research paper format. At the end of the program, the participants presented their research to faculty and other student participants. An abstract was submitted by the coach to a national or regional conference for presentation/publication. The college coach was the abstract’s first author, and the high school students were co-authors.

## Recruitment of College Coaches

The college coaches complete an online application, and those who satisfy the eligibility criteria are selected after an interview process. The eligibility criteria for both programs include a minimum GPA of 3.0 and an essay describing their interest in the program. The coaches do not pay any tuitions and are paid a stipend for the duration of the summer program. The program has been sustainable because 80–85% of high school students pay tuition, and the rest get full scholarships (pay no tuition). As part of both on-site and online programs, one college or medical student coach is assigned to 5–6 high school students, and each coach guides their group on how to do a literature search on a controversial topic. The coaches’ responsibilities are summarized in Table 1.

**Table 1:**
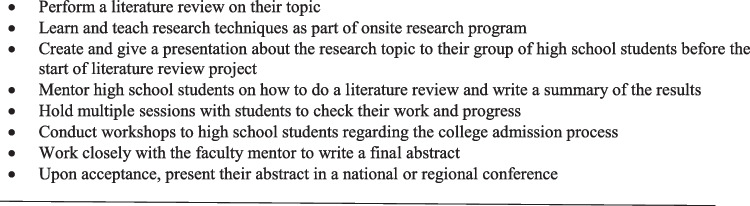
Coach’s responsibilities/activities

Prior to the start of the session, the coaches attend an orientation workshop conducted by the faculty leader, who served as the faculty mentor throughout the program. The guidance provided to the coaches by the faculty mentor during and after the program is summarized in Table 2. As part of the mentorship, the faculty leader kept in touch with the coaches via phone or email. In this article, we specifically focus on the status of the coaches in terms of abstract submission as well as their educational and career outcomes in 2023.

**Table 2:**
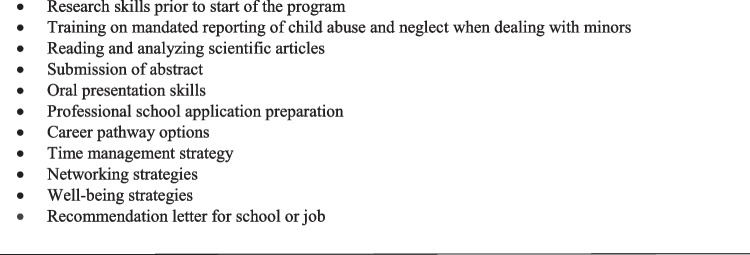
Summary of guidance offered to the coaches through individualized mentorship by faculty mentor(s)

## Results

A total of 54 projects were completed by 54 groups of high school students mentored by 45 coaches between 2012 and 2019 as part of both the on-site and online research programs. Five of the college students served as a coach for the program more than once. The topics of the projects’ topics encompassed various medical specialties and included literature reviews on controversial topics or health disparities. Each year, the abstracts were submitted to the Western American Federation of Medical Research, and all were accepted for publication/presentation. The coaches were the first author and presented the abstract.

**Fig. 1:**
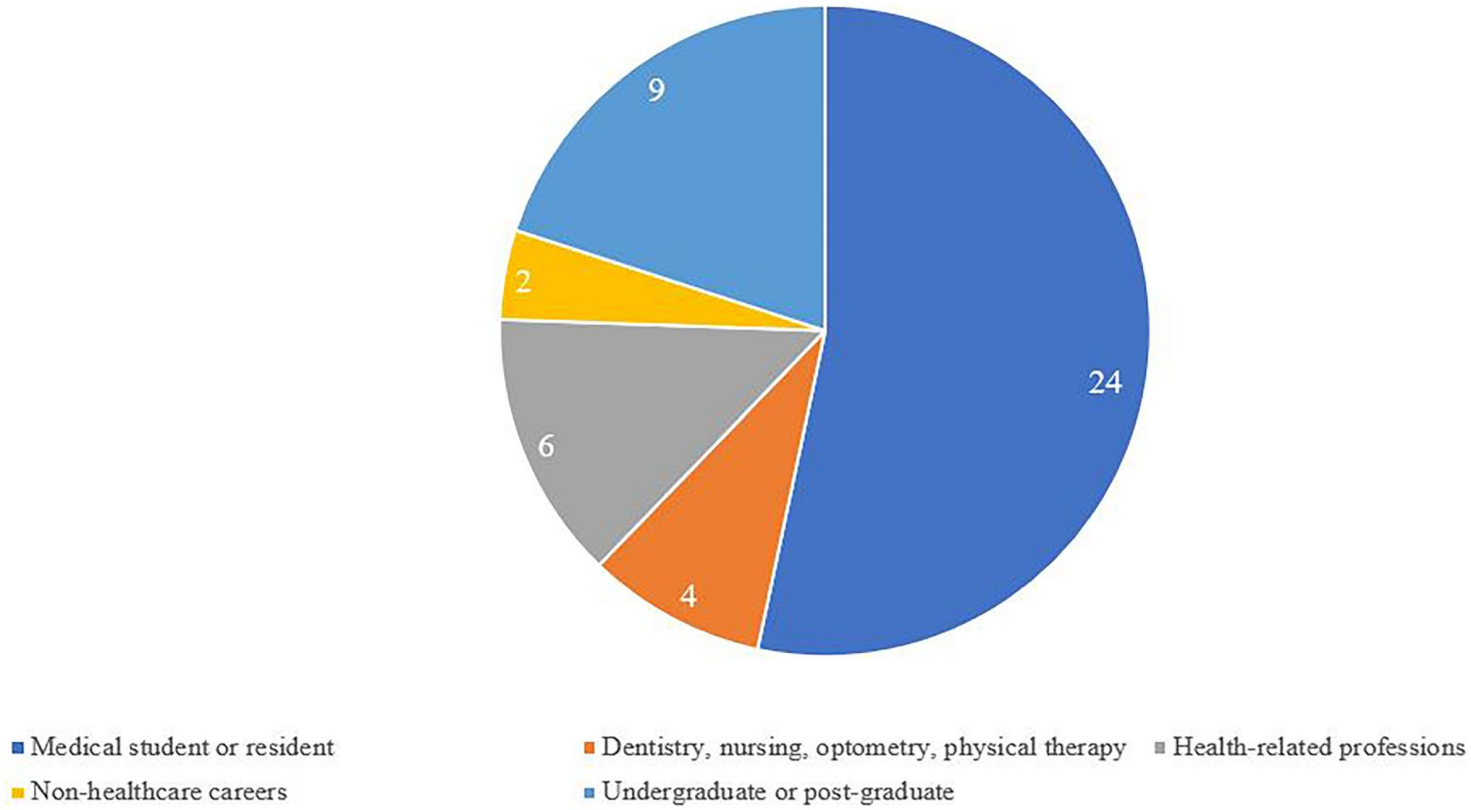
Educational follow-up of the 2012–2019 Summer Research Program college coaches in 2023

Of 45 college coaches, 18 (40%) had participated in our other pipeline programs as high school students before coaching as a part of the research programs, and the rest were new to our program as coaches. On follow-up in 2023, of 45 coaches, 36 (80%) had graduated from college and were either working or attended a health professional school. Of these 36 coaches, 24 (67%) were either attending or had graduated from medical school, four (11%) were attending or had graduated from another type of health-related professional school such as dentistry, or physical therapy, or optometry, and six (17%) were working in a healthcare setting or research field. Of 45 coaches, nine are still completing their undergraduate or graduate degrees in a science-related field. Only two coaches were in non-healthcare–related professions (Fig. 1). In 2023, of 17 URM who completed our program, eight (47%) were in medical school or residency, five (29%) were attending another health professional school or were working in a healthcare setting, and four (24%) were completing college or postgraduate studies. Of 28 females, 18 (64%) were attending medical school, 22% were attending or graduated from other health professional schools or were working in a healthcare setting, and 14% were still in college or postgraduate school.

## Discussion

A few studies have reported that underrepresented postgraduate students in STEM fields publish their research at significantly lower rates compared to non-URMs, putting them at a disadvantage when applying for post-doctorate or faculty positions [14]. Our program was very successful as all the 45 college coaches submitted a total of 54 projects to a regional meeting as the first authors and 100% of the abstracts were accepted for publication. The coaches’ continuous communication with their faculty mentor provided them with foundational knowledge regarding the research-to-publication process and guidance on leading their team of students to achieve their research project goals. The high sense of self-efficacy that developed because of their success in abstract publication and the faculty leader’s continuous mentorship [15] gave the coaches more confidence in persevering in their pathway to enter health and science careers. Comparing the distribution of our summer research program participants and the matriculants of the US medical schools in 2022, our program had a higher percentage of Hispanics/Latinx. Our program comprised 26.7% Hispanics or Latinx and 8.9% Black or African American compared to 6.5% Hispanics/Latinx and 9.3% Blacks/African Americans enrolled in US medical schools, respectively [16]. All the URM coaches who had applied to a health professional school were enrolled or graduated from a healthcare professional school, including medical school, dental school, nursing school, or school of physical therapy. Our female coaches had a higher rate of acceptance to medical school (64%) compared to the overall national acceptance rate of female students who applied to medical school in 2022 (43%) [16].

We believe that our program’s success in meeting our objectives of abstract publication and presentation by the coaches as well as entry to a health professional school was multifactorial. The structural diversity of our program as well as the individualized and goal-oriented mentorship [17] contributed to creating a comfortable space for the coaches that led to the development of self-efficacy skills and allowed the underrepresented minority and female coaches to build a foundation for their future in health or science fields [15]. In addition, the cascading nature and near-peer mentorship model contributed to the success of our program. Overall, 40% of the college coaches for our program had participated as either high school students or a mentor in our enrichment programs. The cyclic nature of learning, mentoring, and advancing health and science careers has been described as one of the advantages of the cascading and near-peer mentorship model [12, 18].

The limitations of this report include the lack of a control group and the small sample size. We cannot conclude that our program was the only factor that resulted in the matriculation of the majority of the students in health professional schools, as many other factors are involved. We are not sure if a group of students with the same academic background and personal characteristics who do not attend our research program would do as well in terms of persisting on their pathway in choosing a career in health sciences. In addition, because of the small sample size, we could not compare the outcome based on gender and ethnicity. It is unclear if the students enrolled in the program will continue to do research as a part of their careers. Additional studies are needed to determine the long-term impact of our program on the coaches’ participation in research. Our preliminary results describe one effort that other institutions can consider in inspiring and preparing college students to follow their career interests in healthcare.

## Data Availability

The data that support the findings of this study are available on request from the corresponding author. The data are not publicly available due to privacy or ethical restrictions.
